# Automated optimization of the solubility of a hyper-stable α-amylase

**DOI:** 10.1098/rsob.240014

**Published:** 2024-05-15

**Authors:** Montader Ali, Matthew Greenig, Marc Oeller, Misha Atkinson, Xing Xu, Pietro Sormanni

**Affiliations:** ^1^ Yusuf Hamied Department of Chemistry, University of Cambridge, Cambridge CB2 1EW, UK; ^2^ Proteomics and Signal Transduction, Max Planck Institute of Biochemistry, Martinsried 82152, Germany

**Keywords:** protein design, enzyme optimization, protein solubility

## Abstract

Most successes in computational protein engineering to date have focused on enhancing one biophysical trait, while multi-trait optimization remains a challenge. Different biophysical properties are often conflicting, as mutations that improve one tend to worsen the others. In this study, we explored the potential of an automated computational design strategy, called CamSol Combination, to optimize solubility and stability of enzymes without affecting their activity. Specifically, we focus on *Bacillus licheniformis* α-amylase (BLA), a hyper-stable enzyme that finds diverse application in industry and biotechnology. We validate the computational predictions by producing 10 BLA variants, including the wild-type (WT) and three designed models harbouring between 6 and 8 mutations each. Our results show that all three models have substantially improved relative solubility over the WT, unaffected catalytic rate and retained hyper-stability, supporting the algorithm’s capacity to optimize enzymes. High stability and solubility embody enzymes with superior resilience to chemical and physical stresses, enhance manufacturability and allow for high-concentration formulations characterized by extended shelf lives. This ability to readily optimize solubility and stability of enzymes will enable the rapid and reliable generation of highly robust and versatile reagents, poised to contribute to advancements in diverse scientific and industrial domains.

## 1. Introduction

Enzymes play crucial roles in biotechnology and find endless industrial applications, where they are harnessed to facilitate a wide range of processes [1]. Their relevance lies in not only catalytic activity but also versatility to operate under diverse conditions. Resilience to thermal, physical and chemical stresses is thus a central consideration for the development of enzymatic biocatalysts [2]. Such stresses can lead to unfolding or aggregation, which hampers activity and product shelf-life. The biophysical properties that underpin this resilience are conformational stability and solubility. Conformational stability is defined as the free energy difference between the native functional state and unfolded states, and in this work, we use the thermodynamic definition of solubility in terms of critical concentration [3]. Together, these properties define colloidal stability, which determines aggregation propensity and the long-term integrity of a formulation [4]. Stability and solubility also correlate with high production yield, the possibility of obtaining high-concentration formulations and long product shelf lives [1,2,5].

Computational tools for protein engineering allow for the rapid, inexpensive and material-efficient screening and optimization of biophysical properties [6]. In recent years, the reliability of these tools has substantially improved, and fully automated methods have been developed to identify point mutations or combinations of mutations likely to improve stability or other properties [2]. For example, tools developed to increase the conformational stability of proteins and enzymes (PROSS, FRESCO, FireProt, etc.), as well as to engineer functionality (FuncLib) have been used to successfully design optimized enzyme variants with improved activity or stability [2]. Notwithstanding these successes, most of these approaches focus on optimizing a single biophysical trait. The key challenge with obtaining proteins that have favourable activity as well as high stability and solubility is that these molecular traits are often conflicting: mutations that improve one trait tend to worsen the other [7]. For example, Broom *et al*. showed that 10 of 11 protein-stabilizing algorithms were successful at predicting stability-increasing mutations, but that these mutations hindered the solubility of the protein [8]. To address this bottleneck, we introduced the CamSol Combination pipeline for the simultaneous optimization of stability and solubility, or of one of these traits without affecting the other. However, the applicability of this approach was demonstrated only for antibodies, and that success strongly relied on large, bespoke, antibody-specific alignments [5].

Here, we extend the applicability of this approach by demonstrating a successful application on enzymes, specifically on *Bacillus licheniformis* α-amylase (BLA). BLA, an enzyme that cleaves starch into smaller carbohydrate units, is indispensable across diverse industries such as food processing, biofuel production, pharmaceuticals and textiles [9]; the family of α-amylases has been reported to account for about 25–30% of the enzyme market [10]. Notably, BLA has exceptional thermostability—exemplified by its starch-liquefying capacity at 110°C [11]—but nevertheless, persistent efforts have been dedicated to enhancing its biophysical characteristics [12].

The growing demand for enzymes across a variety of industries has brought the developability aspects of these proteins to the forefront of protein engineering efforts [13]. Improving stability and solubility is a key focus in these endeavours. Given the challenge of optimizing both properties without compromising enzymatic activity, coupled with the time-consuming and costly nature of large-scale laboratory screening campaigns that also pose environmental concerns, there is a prominent need for fast and resource-efficient computational pipelines to automate the optimization process.

## 2. Results

**Figure 1.**
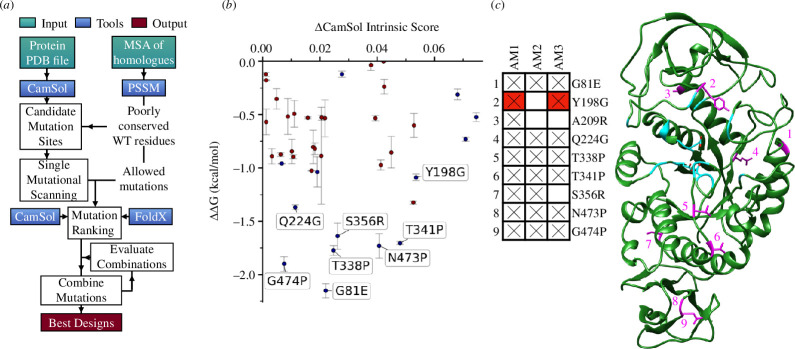
CamSol Combination optimization of BLA. (*a*) Flowchart of the CamSol Combination pipeline, highlighting the main steps to generate the final optimized models (output in red). (*b*) Results of the single mutational scanning step for BLA. Scatter plot of predicted solubility versus stability change for all shortlisted point mutations. Error bars are standard deviations from three FoldX runs. Labelled mutations are those contained in the best-returned models selected for experimental validation; blue points correspond to different mutations also at these sites, while red points are mutations at different sites. (*c*) Table illustrating the mutations contained in the selected models, and structure of *B. licheniformis* α-amylase (BLA, PDB ID: 1BLI) used as input. Y198G is in red background because it is present in the returned models, but it was removed from the models experimentally tested (see text). Active site residues (listed in electronic supplementary material, table S1) are coloured in cyan, and sites mutated in the selected models are in magenta (numbered as in the table).

The CamSol Combination method is aimed at optimizing the solubility of a target protein while retaining or improving its conformational stability, without impacting functionality. The inputs are the wild-type (WT) structure or structural model and a corresponding multiple sequence alignment (MSA) to extract phylogenetic information. The output consists of several designs harbouring single or multiple mutations predicted to improve solubility and stability. The pipeline is described in detail by Rosace *et al*. [5]. and illustrated in figure 1*a*.

Here, we applied the automated optimization pipeline to the hyper-stable BLA enzyme (PDB ID: 1BLI). To obtain a suitable MSA for the BLA sequence, we relied on the widely used automated HHblits method [14]. To reduce the chances of compromising enzyme activity, we could have excluded active site residues and their neighbourhood from the list of possible mutation sites. However, to test for a more generic case in which such information may not be available, we decided not to exclude any residues from the calculations to see whether the conservation of such residues, as inferred from the input MSA, is enough to prevent them from being mutated.

First, residue substitutions allowed by the position-specific scoring matrix (PSSM) calculated from the MSA are singly modelled at sites automatically identified based on their contribution to solubility, conservation and solvent exposure [5]. The PSSM calculated from the input MSA is depicted in electronic supplementary material, figure S1, with those WT residues that have a negative enrichment coloured red. These are flagged as possible mutation sites. The CamSol structurally corrected and intrinsic profiles are shown in electronic supplementary material, figure S2, together with candidate mutation sites identified from these profiles as likely to improve solubility when mutated. PSSM-allowed mutations at these sites that increase the intrinsic CamSol score (predicted solubilizing) and decrease the FoldX ΔΔG (predicted stabilizing) are shortlisted. This procedure selected 39 mutations at 21 distinct sites from 66 identified sites (figure 1*b*; electronic supplementary material, table S1). Shortlisted mutations are then ranked and combined in multiple designs, with up to 12 mutations each, to form the best-returned models (electronic supplementary material, table S2). We selected three amylase models (AM) for experimental testing (figure 1*c*), the overall best design with nine mutations, here denoted as AM1, and two high-ranking models with seven mutations, denoted AM2 and AM3 (electronic supplementary material, table S2).

**Figure 2.**
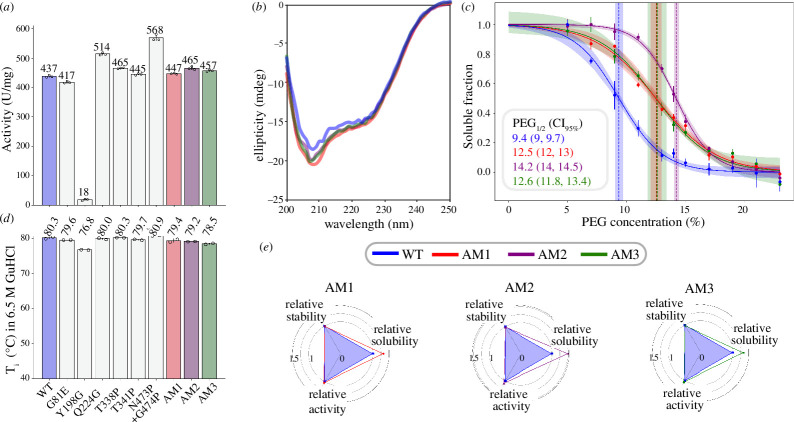
Experimental characterization of amylase variants, both single or double mutants, and models rationally designed with mutation combinations. (*a*, *d*) Bar plots of the activity (*a*) and temperature of inflection (*T*_i_ in 6.5 M GuHCl (*d*) of each variant (x-axis). Bar heights are means (also shown as value labels), error bars are standard deviations and points are the outcome of individual experiments. (*b*) Circular dichroism (CD) spectra of WT and AM (see legend). (*c*) Polyethylene glycol (PEG) precipitation assay carried out on the WT and models (see legend), the shading represents the 95% confidence interval (CI_95%_) on the fit, and dashed vertical lines are the fitted PEG_1/2_ values with their CI_95%_. (*e*) Radar charts summarizing the measured biophysical properties of each designed BLA model (title) relative to the WT (blue triangle).

We first produced single mutants to high purity (electronic supplementary material, table S3 and figure S3) and measured their catalytic activity (figure 2*a*; electronic supplementary material, figure S5). The G81E and Q224G variants had activities comparable to that of the WT, while all proline mutants (T338P, T341P, N473*p*+G474P) had a marginally increased activity over that of the WT (figure 2*a*). Conversely, Y198G had substantially decreased activity. We also measured conformational stability with heat denaturation using nano differential scanning fluorimetry, a technique commonly used for the determination of the thermal stability of enzymes. However, as BLA is a hyper-stable enzyme, to observe a melting transition we had to carry out heat denaturation experiments in 6.5 M guanidine hydrochloride (GuHCl) following overnight incubation. Except for Y198G, which had a temperature of inflection (*T*_i_) 3.5°C lower than that of the WT, all other single mutants had a *T*_i_ within ±0.7°C of that of the WT (figure 2*b*; electronic supplementary material, table S4).

Based on these results, we decided to remove the Y198G mutation from the AM1 and AM3 models. We produced the three models to high purity (electronic supplementary material, table S3 and figure S3) and carried out the same activity and stability measurements (electronic supplementary material, table S4). We find that all models have activity at par or slightly better than that of the WT (figure 2*a*) and comparable stabilities (figure 2*b*). We further carried out measurements of circular dichroism (CD) and found that models and WT produced highly similar spectra typical of ⍺-helix structures (figure 2*c*).

We then sought to measure relative solubility using the well-established PEG precipitation technique (see §4). The solubility curves from the PEG precipitation assay produced PEG_1/2_ values significantly larger for the designed AM than for the WT, indicating a much-improved relative solubility (figure 2*d*). Specifically, the WT has a PEG_1/2_ of 9.4% (weight/volume), while the most soluble model, AM2, has 14.2% and AM1 and AM3 have comparable PEG_1/2_ of around 12.5%.

## 3. Discussion

The automated CamSol Combination optimization pipeline has previously been used to successfully optimize the solubility and conformational stability of antibodies [5]. Here, we sought to determine whether the method was similarly effective when applied to enzymes. To do this, we used an MSA automatically obtained from our target sequence using the popular HHblits algorithm [15] instead of the antibody-specific alignments used previously [5]. We employed BLA, a hyper-stable ⍺-amylase enzyme, as a proof of concept.

Ten variants of BLA were produced in this work: the WT, five single mutants, one double mutant (N473P + G474P) and three models with different combinations of mutations returned by the algorithm. We measured apparent thermal stability in 6.5 M GuHCl (*T*_i_) and catalytic activity (U/mg) for all variants. Conversely, PEG precipitation relative solubility measurements were performed only on the three models and the WT, since this assay is material-intensive and unlikely to reliably detect the effect of a single mutation [5].

The measured difference in PEG_1/2_ between the WT and the most soluble model AM2 (ΔPEG_1/2_ = 5.2%) corresponds to a very substantial improvement in relative solubility, given the dynamic range of this assay [16]. To put this number in context, the ΔPEG_1/2_ between MEDI578 and MEDI1912 is 4.5%, as reported in Ref [17]. These are two well-studied model monoclonal antibodies, respectively, examples of a soluble, developable mAb and its affinity-matured counterpart, which has lost its developability potential and has a compromised solubility [18].

Preferably, enzymes’ active sites and their neighbours should be marked as ‘excluded residues’ in the input of the CamSol Combination pipeline. However, in this study, we investigated whether the MSA conservation of functionally relevant residues would be enough to prevent the algorithm from mutating them. Our results confirmed that none of the 66 candidate mutation sites automatically identified were on the active site. However, one site (Y198) is adjacent to the sugar-binding residue M197 (highlighted in figure 1*c*), suggesting that the Y198G mutation may affect activity, as confirmed by our data (figure 2*a*), for example by increasing local flexibility. Therefore, when functionally relevant residues are not known, it is advisable to test single mutations individually before combining them in models, as we have done in this work.

All three selected models of BLA exhibited substantially improved relative solubility without showing any detrimental effect on conformational stability and activity (figure 2*e*; electronic supplementary material, table S4). Interestingly, the automated design pipeline did not find mutations that further increased stability, in agreement with the fact that BLA is already hyper-stable with no or limited room for further improvement. Conversely, the selected mutations had a substantial effect on relative solubility and, crucially, left stability and activity unaffected. A similar observation was made when applying CamSol Combination to the Fv region of the therapeutic antibodies adalimumab and golimumab [5]. In a study that characterized the biophysical landscape of clinical-stage antibodies, adalimumab did not show any self-association or cross-reactivity flags, while golimumab had several [19]. In agreement with this fact, the CamSol Combination-optimized designs of golimumab showed large improvements in relative solubility and minor improvements in stability, while the opposite was observed for adalimumab [5]. Therefore, it is expected that proteins with already optimal properties will show smaller improvements than those with more modest solubility or stability.

The effectiveness of CamSol Combination on BLA indicates that it can serve as a valuable tool for optimizing proteins and enzymes. Building on this proof of concept, future applications of the algorithm can rely on the suggested mutations to enhance stability and solubility of proteins of interest without compromising functionality. Although this method was applied to BLA, an enzyme with superior stability to most proteins, many reporter proteins used in diagnostic assays have substantially lower stabilities, thus providing further opportunities for optimization. Overall, our findings show that this approach can facilitate enzyme development and may serve a variety of important applications in research, diagnostics and industrial biotechnology.

## 4. Methods

### 4.1. CamSol combination of BLA

The BLA sequence was extracted from the *seqres* field of the PDB file 1BLI (chain A). This sequence was used as input to HHblits, which was run with the command *hhblits -cpu 16 -i 1bli_A.fa -o 1bli_id95_cov75 -oa3m 1bli_id95_cov75_hhblits_alignment.a3m -ohhm 1bli_id95_cov75_hhblits.hhm -n 3 -id 95 -qid 0 -diff inf -cov 75*. Alternatively, the user-friendly HHblits web server could be used to obtain the same alignment [14]. The generated alignment file together with the BLA structure (PDB file 1BLI) was used as input for the CamSol Combination algorithm. Cysteine, methionine and asparagine amino acids were excluded from the list of possible target substitutions, which is the default behaviour, as these residues can lead to known liabilities like disulphide-mediated dimerization, oxidation, and glycosylation or deamidation. No residue position was excluded from the list of candidate mutation sites. The default ‘alignment frequency strong filter’ option was used, meaning that the mutational space was restricted to residues that are enriched in the PSSM and more enriched than the WT residue at that position (log-likelihood > 0 and Δlog-likelihood > 0). The algorithm was run by allowing a maximum of nine mutations in combination.

### 4.2. BLA production and purification

The genes encoding the BLA WT, AM1, AM2 and AM3 were cloned in the plasmid PET-29(b+), including a C-terminal 6× His-Tag and a leading NSP4 sequence [20] (electronic supplementary material, table S5), which is cleaved upon translocation to the periplasm. Single mutants were obtained with mutagenesis using the QuikChange Lightning (Agilent RUO 210519) kit, and the primers employed were obtained from the Agilent PrimerDesign Program webserver. Polymerase chain reaction was performed in a Bio-Rad C1000 Touch machine followed by Dpn I digestion of the WT gene [20]. All BLA variants were expressed using the *E. coli* BL21 DE3 (PlysS) cell line. Cells were grown in a small culture overnight at 37°C in the presence of 50 μg ml^−1^ kanamycin (Thermo Fisher Scientific, USA, cat. 15160054) and 34 μg ml^−1^ chloramphenicol (Thermo Fisher Scientific, cat. B20841.22). One litre of LB media with the same antibiotics was inoculated at 0.1 OD600 nm and grown at 37°C until 0.8 OD600 nm. Induction was done with IPTG at a final concentration of 0.05 mM. Overnight expression was performed at 16°C. After 24 h, a second round of induction at 0.05 mM IPTG was performed, including the addition of lyophilized lysozyme (Thermo Fisher Scientific, cat. 89833) to a final concentration of 0.3 mg ml^−1^. The lysozyme is added to assist in the breakdown of the peptidoglycan layer of the *E. coli* outer membrane and to facilitate purification from the media. The cells continued to grow overnight at 16°C before purification from media the following day.

To capture the His-tagged enzyme, AmMag Ni Magnetic Beads (GenScript, cat. L00776) were added to the media at a concentration of 0.2 ml per litre of cell culture. These were incubated at 16°C for 2 h, shaking at 200 r.p.m. Magnetic beads were collected, washed in PBS and loaded onto the GenScript AmMag SA Plus purification system (GenScript, cat. L01013), where the beads were washed in PBS with 4 mM imidazole pH 7.4, and the protein was eluted and collected in PBS fractions with 40- and 200 mM Imidazole. The proteins were further purified via gel filtration using a Superdex 200 column (Cytiva, 17-5174-01). Protein concentrations were determined by absorbance measurements at 280 nm using theoretical extinction coefficients calculated with the Expasy ProtParam web server. The mass of each protein purified was confirmed by comparing the theoretical molecular weight to that obtained from liquid-chromatography mass spectroscopy using VION (Waters).

### 4.3. Nano differential scanning fluorimetry

The melting temperature of the enzymes was measured with Tycho NT.6 (Nanotemper). Intrinsic fluorescence of tryptophan and tyrosine residues was recorded at 330 and 350 nm with a fixed 30°C min^−1^ temperature ramp from 35 to 95°C. The ratio of fluorescence intensity (350/330 nm) and the inflection temperature *T*_i_ was calculated by the software on the Tycho NT.6 instrument (electronic supplementary material, figure S4).

As the melting temperature of BLA is above 95°C, to obtain the melting curves, all variants were incubated in guanidine HCl (Thermo Fisher Scientific, cat. 24110) at final concentrations of 0.05 mg ml^−1^ protein and 6.5 M guanidine HCl. All samples were incubated overnight in duplicates before taking one heat denaturation reading per replicate. Melting curves are reported in electronic supplementary material, figure S4.

### 4.4. Circular dichroism

Far-ultraviolet (UV) CD spectra of the BLA variants were recorded using a Chirascan Applied Photophysics spectropolarimeter equipped with a Peltier holder, using a 0.1 cm path length quartz cuvette. Samples contained 0.224 mg ml^−1^ protein in PBS. The far-UV CD spectra of all variants were recorded from 200 to 250 nm at 25°C, and the spectrum of the PBS buffer was systematically subtracted from the spectra of all enzymes to yield the plots in figure 2*c*.

### 4.5. Enzymatic activity measurements

Amylase activity assays were performed using the Amylase Activity Assay Kit (Sigma-Aldrich, cat. MAK009) on all the amylase variants. The assay was performed on Greiner 384 well F-bottom non-binding plate (Greiner BIO-ONE, cat. 781900) and measured on a CLARIOSTAR plate reader (BMG). For each BLA variant, the concentration was prepared to be 1 nM in 200 µl. All dilutions were performed in Amylase Activity Buffer (Sigma-Aldrich, cat. MAK009A). A volume of 15 µl of each BLA variant at 1 nM was added to the 384 well plate. The plate was inserted into a CLARIOstar plate reader, where 30 µl of Amylase Substrate Mix (Sigma-Aldrich, cat. MAK009B) was injected into every well. Immediately after the automated injections, the absorbance at 405 nm (A405) was measured every 25 s for 55 min for all 10 variants. The nitrophenol standard for colorimetric detection was set up as reported by the assay kit, using the supplied nitrophenol standard (Sigma-Aldrich, cat. MAK009D). The amount of p-nitrophenol produced was calculated using the standard plot (electronic supplementary material, figure S5*b*). The amount of p-nitrophenol produced was then used to calculate the activity (U/mg) of each variant as


$$amylase\;activity=\frac{B\times D}{T_{rxn}\times V}$$

where *B* is the amount (nmole) of nitrophenol generated between the initial and final time (*t*_initial_ and *t*_final_), *D* is the sample dilution factor. The reaction time *T*_rxn_ is the difference between *t*_final_ and *t*_initial_ and *V* is the sample volume (ml) added to each well. The amylase activity (nmole min^−1^ ml^−1^) was then used to calculate the reported U mg^−1^ value.

### 4.6. PEG precipitation

The relative solubility of each variant was measured with PEG precipitation, using an adaptation of the protocol recently introduced by Oeller *et al*. [16]. Briefly, PEG solutions at concentrations ranging from 0% to 30% (w/v) were prepared from a 50% PEG6000 (Sigma-Aldrich, cat. 81260) stock solution in PBS at pH 7.4 (pH re-adjusted after adding PEG, and on the day of the experiment). Then, an aliquot of protein from a stock at 3 mg ml^−1^ was mixed with each PEG solution to obtain a final protein concentration of 1 mg ml^−1^. The assay was performed in 384 well plates sealed with aluminium plate sealers to limit evaporation (Thermo Fisher Scientific). Plates were incubated at 4°C for 48 h and centrifuged at 4°C at 6000*g* for 2 h. After that, the supernatants of each well were transferred to a new UV-transparent plate. Supernatant concentrations were measured on the CLARIOstar plate reader using blank absorbance values of 280 nm. Each condition was made in quadruplicate. The soluble concentration obtained was normalized to the 0 PEG concentration and fitted to a sigmoid function to obtain the PEG_1/2_ value as a proxy for relative solubility. The error on the PEG_1/2_ was estimated by a 95% confidence interval analysis using bootstrapping.

## Data Availability

All data needed to evaluate the conclusions in this article, or that are necessary to interpret, verify and extend the research in the article are present in the paper and/or the electronic supplementary material [21]. Additional details are available from the corresponding author on request. The CamSol Combination method is made available to the academic community as a web server at https://www-cohsoftware.ch.cam.ac.uk/index.php. To access the software, users need to register a free account and log in.
